# Differential effects of antigens from *L. braziliensis *isolates from disseminated and cutaneous leishmaniasis on *in vitro *cytokine production

**DOI:** 10.1186/1471-2334-6-75

**Published:** 2006-04-25

**Authors:** Paulo TG Leopoldo, Paulo RL Machado, Roque P Almeida, Albert Schriefer, Angela Giudice, Amélia Ribeiro de Jesus, John L Ho, Luiz Henrique Guimarães, Olívia Bacellar, Edgar M Carvalho

**Affiliations:** 1 Departamento de Fisiologia, Universidade Federal de Sergipe, Aracaju, Sergipe, Brazil; 2 Serviço de Imunologia, Hospital Universitário Prof. Edgard Santos, Instituto de Investigação em Imunologia (iii), Universidade Federal da Bahia, Salvador, Bahia, Brazil; 3 Departamento de Biointeração, Instituto de Ciências da Saúde, Universidade Federal da Bahia, Salvador, Bahia, Brazil; 4 Division of International Medicine and Infectious Diseases, Department of Medicine, Weill Medical College of Cornell University, New York, NY, USA

## Abstract

**Background:**

Disseminated leishmaniasis is an emerging infectious disease, mostly due to *L. braziliensis*, which has clinical and histopathological features distinct from cutaneous leishmaniasis.

**Methods:**

In the current study we evaluated the *in vitro *production of the cytokines IFN-γ, TNF-α, IL-5 and IL-10 by peripheral blood mononuclear cells (PBMC) from 15 disseminated leishmaniasis and 24 cutaneous leishmaniasis patients upon stimulation with *L. braziliensis *antigens genotyped as disseminated leishmaniasis or cutaneous leishmaniasis isolates.

**Results:**

Regardless of the source of *L. braziliensis *antigens, PBMC from cutaneous leishmaniasis patients produced significantly higher IFN-γ than PBMC from disseminated leishmaniasis patients. Levels of TNF-α by PBMC from cutaneous leishmaniasis patients were significantly higher than disseminated leishmaniasis patients only when stimulated by genotyped cutaneous leishmaniasis antigens. The levels of IL-5 and IL-10 production by PBMC were very low and similar in PBMCs from both disseminated leishmaniasis and cutaneous leishmaniasis patients. The immune response of each patient evaluated by the two *L. braziliensis *antigens was assessed in a paired analysis in which we showed that *L. braziliensis *genotyped as disseminated leishmaniasis isolate was more potent than *L. braziliensis *genotyped as cutaneous leishmaniasis isolate in triggering IFN-γ and TNF-α production in both diseases and IL-5 only in cutaneous leishmaniasis patients.

**Conclusion:**

This study provides evidence that antigens prepared from genotypically distinct strains of *L. braziliensis *induce different degrees of immune response. It also indicates that both parasite and host play a role in the outcome of *L. braziliensis *infection.

## Background

*Leishmania *are obligate intracellular protozoa transmitted to mammals by infected sand flies. Human infection ranges from asymptomatic to tegumentary and visceral disease, with disfiguring and fatal outcomes in the most severe cases [1]. American tegumentary leishmaniasis (ATL) presents a spectrum of clinical manifestations including cutaneous (CL), mucosal (ML), disseminated (DL) and diffuse cutaneous leishmaniasis [2,3]. DL caused by *L. braziliensis *is an emerging infectious disease mainly observed in Northeastern Brazil. DL is characterized by numerous acneiform, papular, nodular and ulcerated skin lesions, distributed in two or more body parts [4-7]. Most importantly, compared to only 3% among CL patients develop mucosal leishmaniasis, up to 28% of DL cases develop mucosal lesions, and the majority manifest mucosal disease concurrent with cutaneous lesions at disease presentation [7].

Previous studies with small numbers of DL patients have suggested that they often present a negative *Leishmania *skin test and that their lymphocytes produce lower levels of Th1 cytokines upon antigen stimulation than those of CL individuals [7]. In addition to the roles of host genetics and immune responses, evidence has been accumulated on the intra-species variability among *Leishmania sp*, and its importance in the development of different clinical forms of leishmaniasis [8-12]. Analyses employing techniques like multilocus enzyme electrophoresis (MLEE) and rRNA gene internal transcribed spacers PCR-RFLP (ITR) showed a high degree of polymorphism between isolates of *L. braziliensis *from different regions. Moreover, our recent analyses of the populations of parasites in the ATL endemic area of Corte de Pedra (CP), northeastern Brazil, revealed polymorphism among *L. braziliensis *isolates and associations between genotypes and disease outcome [8].

In this work to discern whether parasite factors influence outcome of infection towards one of these two forms of ATL, we assessed the production of IFN-γ, TNF-α, IL-5 and IL-10 through the stimulation *in vitro *of peripheral blood mononuclear cells (PBMC) from DL and CL patients with *L. braziliensis *antigens isolated from DL and CL patients.

## Methods

### Subjects

The patients enrolled in this study were recruited at the Corte de Pedra Health Post, situated in the southeast region of the State of Bahia, Brazil. The inclusion criteria were diagnosis of CL and DL with the presence of active skin lesions, and age from 10 to 60 years. The exclusion criteria were pregnancy, AIDS and other immunosuppressive conditions, as well as the patient's desire of being excluded from the study. Participants of this study included 15 DL patients presenting with 10 or more mixed-type lesions (acneiform, papular, nodular and/or ulcerated) in two or more body parts (head, trunk, arms and legs), and 24 CL patients presenting with ulcerated lesions localized in a single body part. The diagnosis was confirmed by culture identification of *Leishmania *in lesion biopsy or in aspirates, or by histopathology typical of leishmaniasis and a positive *Leishmania *skin test.

This study has been approved by the Ethical Committee of the Hospital Universitário Professor Edgard Santos and informed consent was obtained from all prospectively enrolled patients.

### Parasites

Two promastigotes of *L braziliensis*, one from a patient with CL and the other from a patient with DL, each genotyped by RAPD protocols were used as antigen sources for the *in vitro *cytokine induction assays. The molecular genotyping of parasites was made by four RAPD protocols as previously described [8]. Cutaneous leishmaniasis *L. braziliensis *isolate belonged to CL enriched *L. braziliensis *clade B of CP, while the DL isolate belonged to the DL enriched clade D of CP. Therefore, they are designated as *L. braziliensis *genotyped as CL or DL isolates. In a small number of patients experiments were repeated with two other isolates from different patients with CL and DL. The results were similar to these obtained with the original isolates.

### Isolation and cultivation of L braziliensis

The isolation of *L. braziliensis *was made in Navy McNeal Nicoli (NNN) blood agar overlaid with a modified Liver Infusion Tryptose (LIT) supplemented with 10% heat inactivated fetal bovine serum medium (FCS) (Sigma Chemical Co., St. Louis, MO) from samples obtained by needle aspiration of the skin lesions. The cultures from the biphasic medium were expanded for growth in Schneider's insect medium (Sigma), supplemented with 10% heat inactivated FCS at 25°C.

### Leishmania antigens

Antigens used for *in vitro *stimulation of PBMC came from isolates of *L. braziliensis *from CL and DL patients. Promastigotes in the stationary-phase of growth were washed three times by centrifugation, adjusted to a concentration of 10^9 ^in Phosphate Buffered Saline (PBS), and disrupted by eight repeated cycle of freezing (-70°C) and thawing (37°C), followed by ultrasonication. The supernatants were filtered at 0,45 υM Millipore and stored at -20°C until use. The protein content was determined by the Lowry method [13]. After performing a dose curve response to determine the optimal dose, both antigens were used in the concentration (10 μg/ml).

### Separation and culture of cells

PBMC were obtained from heparinized venous blood from patients with CL and DL by density centrifugation over a gradient of Ficoll-Hypaque (LSM; Organon Teknika corporation, Durham, NC, USA). PBMC adjusted to a concentration of 3 × 10^6 ^cells/ml were suspended in RPMI 1640 medium (GIBCO BRL., Grand Island, NY), 10% heat-inactivated human AB serum, 2 mM L-glutamine, 100 U/ml penicillin and 100 μg/ml streptomycin (GIBCO). The cells were distributed in 24-well plates and stimulated with *Leishmania *antigens (10 μg/ml) and phytohemaglutinin (PHA) (100 μg/ml, Sigma), and then incubated for 72 hr at 37°C in 5% CO_2 _.

### Cytokine determination

The supernatants were harvested and stored at -20°C for cytokine determination [14]. Supernatants of cell cultures from CL and DL patients were assayed for IFN-γ, TNF-α, IL-5 and IL-l0 by sandwich ELISA technique (R & D system, Minneapolis, MN) using a mouse anti-human cytokine monoclonal antibody (mAb) as capture antibody. Purified cytokines at varying amounts were used to derive a standard curve (R & D system, Minneapolis, MN). The sensitivities of the cytokine dosage assays were 8 pg/ml (for IFN-γ), 4.4 pg/ml (for TNF-α), 3.9 pg/ml (for IL-5) and 3.9 pg/ml (for IL-10). IL-4 was not measured because of the low sensitivity of the technique in detecting IL-4 in supernatants of PBMC from humans.

### Statistical analysis

Comparison of the levels of cytokine produced by PBMC from CL and DL patients was made by the Mann-Whitney test. The paired analyses were conducted by Wilcoxon paired non-parametric test; significance was determined as p < 0.05 (two tailed).

## Results

**Figure 1:**
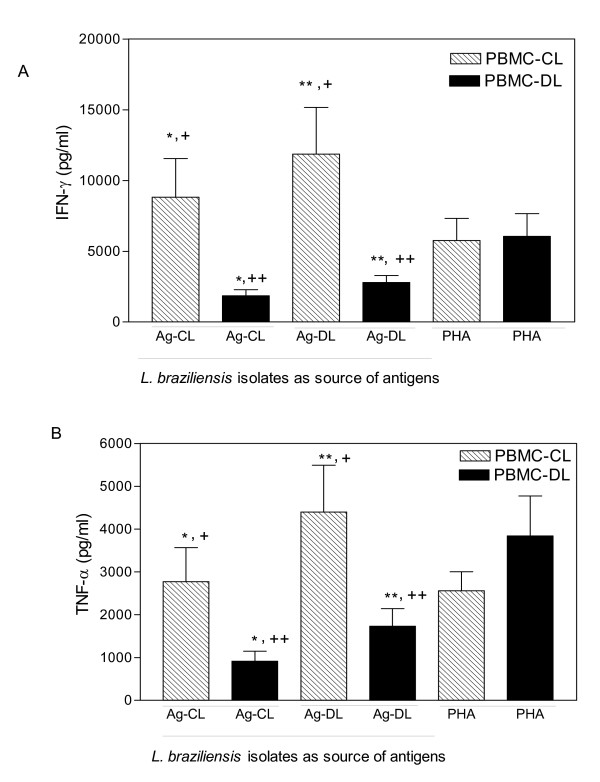
**IFN-γ and TNF-α production induced by antigens from *L. braziliensis *isolates isolated CL or DL cases**. PBMCs from cutaneous leishmaniasis (CL) (hatched bars, n = 24) and disseminated leishmaniasis patients (black bars, n = 15) were stimulated with antigens of *L. braziliensis *genotyped isolate from cutaneous leishmaniasis (Ag-CL) and disseminated leishmaniasis (Ag-DL). Data represent mean ± standard error mean (SEM) of supernatants collected and assayed by ELISA for (***A***) IFN-γ, *P < 0.02 and **P < 0.01, Mann-Whitney test; ^+^P < 0.005 and ^++^P < 0.001, Wilcoxon paired non-parametric test; and for **(B)** TNF-α, *P < 0.03 and **P < 0.8, Mann-Whitney test; ^+^, ^++^P < 0.001, Wilcoxon paired non-parametric test.

The production of IFN-γ and TNF-α by peripheral blood mononuclear cells (PBMC) from CL and DL patients stimulated with antigens from *L. braziliensis *genotyped isolates are shown in figure 1. *In vitro *IFN-γ production by PBMC from CL patients was significantly higher than DL patients regardless of whether the source of antigen was *L. braziliensis *genotyped as a CL or DL isolate (8810 ± 13422 pg/ml versus 1849 ± 1683 pg/ml or 11873 ± 16184 pg/ml versus 2791 ± 1881 pg/ml, respectively; P = 0.02 or = 0.01, respectively) (Figure 1A). TNF-α production by PBMC from CL patients was significantly greater than DL patients in response to *L. braziliensis *antigen from a CL isolate. Even though *L. braziliensis *antigen from a DL isolate triggered higher TNF-α production by CL patients than DL patients (4400 ± 5262 pg/ml versus 1729 ± 1603 pg/ml) the difference did not reach statistical significance (Figure 1B, P = 0.08). The observed finding of higher levels of IFN-γ and TNF-α production by CL patients appeared to be specific because PBMC from CL and DL patients responded to the mitogen, PHA, by producing similar amounts of IFN-γ and TNF-α. We also evaluated whether the response of each patient in the specific disease group was influenced by the source of the *L. braziliensis *antigens. The aim was to compare IFN-γ and TNF-α levels of CL patients to the two *L. braziliensis *antigens and the immune response of DL patients to the two *L. braziliensis *antigens. Using a paired analysis, *L. braziliensis *antigens from a DL isolate compared to a CL isolate triggered significantly greater (P < .05) IFN-γ and TNF-α production by PBMC from both CL and DL patients (Figures 1A e 1B).

**Figure 2:**
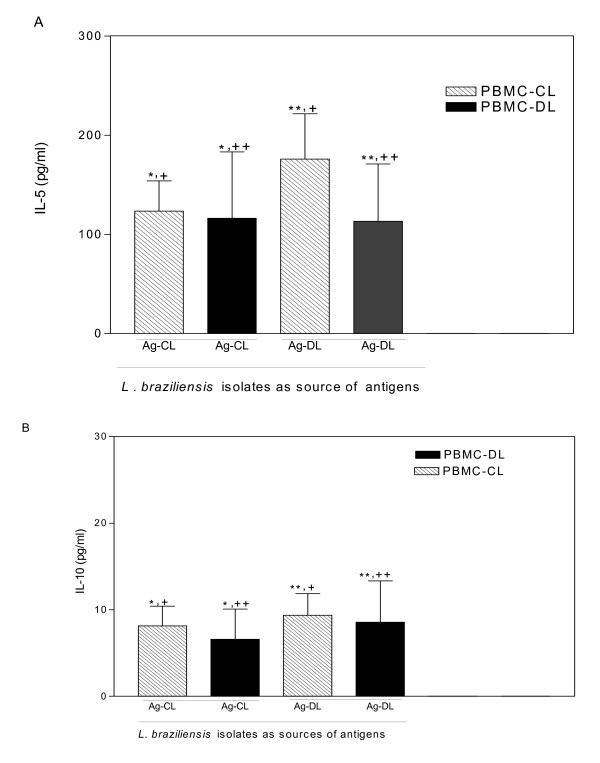
**IL-5 and IL-10 production induced by antigens from *L. braziliensis *isolates isolated CL or DL cases**. Data of cutaneous leishmaniasis patients (hatched bars) and disseminated leishmaniasis patients (black bars) are illustrated as the mean ± SEM of the same supernatants from Figure 1 assayed by ELISA for IL-5 (***A***) *P = 0.07, **P = 0.13, Mann-Whitney test; ^+^P < 0.008 and ^++^P < 0.08, Wilcoxon paired non-parametric test and IL-10 (***B***) *,**P = 0.4, Mann-Whitney test; ^+^P = 0.5 and ^++^P < 0.4, Wilcoxon paired non-parametric test.

We next tested IL-5 and IL-10 from the same supernatant previously assayed for IFN-γ and TNF-α. The levels of IL-5 and IL-10 produced by PBMC from patients with CL and DL were similar (Figure 2A). The response of each patient in the CL and DL groups to the two *L. braziliensis *antigens was also evaluated and showed that *L. braziliensis *antigens from a DL isolate triggered significantly higher IL-5 production than antigens from a CL isolate by PBMC from CL patients. Such a difference was not observed when tested by PBMC from DL patients (Figure 2A). Levels of IL-10 production by PBMCs from CL and DL patients were similarly low in response to antigens from *L. braziliensis *CL isolate (6 ± 5 pg/ml and 9 ± 14 pg/ml (mean + SD)) and *L. braziliensis *DL isolate (8 ± 12 pg/ml and 9 ± 16 pg/ml), P = 0.41 and P = 0.41, respectively (Figure 2B). Over 50% of the CL and DL patients' PBMC cultures had undetectable levels of IL-10.

## Discussion

Disseminated leishmaniasis is an emerging disease predominantly detected in the state of Bahia, Brazil [4,5,7]. Although *L. amazonensis *and *L. braziliensis *have been reported to be associated with DL in the past, only *L. braziliensis *has recently been consistently implicated with this disease [7]. Furthermore, we have recently demonstrated that certain *L. braziliensis *genotypes are more associated with DL in areas of endemicity for ATL [8]. In the current study we evaluated the production of IFN-γ, TNF-α, IL-5 and IL-10 of PBMC from DL and CL patients, showing that DL patients produce less IFN-γ and TNF-α than those with CL upon antigen stimulation, while the two groups of individuals present similar IL-5 and IL-10 secretion. In addition, we found that antigen from parasites of DL origin is a stronger inducer of the above pro-inflammatory cytokines. These data concur with results from previous work in our lab, in which we demonstrated that PBMC from DL stimulated *in vitro *with antigens of *L. amazonensis *produced lower levels of pro-inflammatory cytokines than those of CL individuals [7]. Reinforcing these findings, it has been shown that biopsies from CL patients display a higher intensity of IFN-γ expression than those of DL individuals [15]. While lower levels of IFN-γ and TNF-α may contribute to the dissemination of the disease, they may also play a part in the different clinical aspects of the lesions observed in these two clinical forms of *L. braziliensis*. These pro-inflammatory cytokines may play key roles in the defense mechanism against *Leishmania*, but may also be involved in the formation of the immune mediated ulcers of CL [16,17]. Therefore, in contrast to the CL patients who develop large ulcers with infiltration of lymphocytes in the lesions and absent or few parasites, DL individuals present multiple lesions, frequently over 100, in different parts of their body. These lesions are mostly non-ulcerated and small, and the parasites are more easily documented [5].

Studies performed in Ethiopia on the stimulation of PBMC from CL and diffuse cutaneous leishmaniasis, a disease clinical and pathologically distinct from DL, have shown that PBMC from CL produce more IFN-γ when stimulated with *L. aethiopica *antigen from CL than by antigen from diffuse cutaneous leishmaniasis. Interestingly, lymphocyte proliferation among control individuals of that endemic area was higher in response to CL antigen than diffuse cutaneous leishmaniasis antigen, supporting the idea that differences in the parasites may contribute to the clinical outcome of infection with *L. aethiopica *[18]. Unlike *L. aethiopica*, *L. braziliensis *does not cause diffuse cutaneous leishmaniasis and is predominantly associated with CL and ML diseases, characterized by a strong type 1 immune response. While indicating phenotypic variability among the strains, the observation that DL-borne *L. braziliensis *induced significantly higher levels of IFN-γ and TNF-α in cultures of PBMC from CL patients was still surprising. We offer two possible explanations: (1) a bias in the innate immune response, which might be determining the outcome of DL; and (2) an over modulation of production of these cytokines by regulatory T lymphocytes in DL individuals with carryover effects to the *in vitro *evaluations. Both possibilities are currently being evaluated.

IFN-γ and TNF-α are important cytokines for the outcome of infection in the experimental models of leishmaniasis. Type 1 immune responses are associated with control of infection, while type 2 leads to susceptibility for *Leishmania *sp [19]. In humans, low IFN-γ production has been associated with parasite dissemination, as exemplified by VL and diffuse cutaneous leishmaniasis [20-23], in which IL-10 acts to down regulate IFN-γ [24]. In contrast to what has been observed in VL and diffuse cutaneous leishmaniasis, we found neither a type 2 immune response nor an increase in IL-10 levels in DL patients [23,25]. However, it was clear the type 1 immune response is impaired in DL patients.

## Conclusion

This study showed that regardless of the source of antigens, CL patients produce more IFN-γ than DL patients. Antigens from *L. braziliensis *genotyped as a DL isolate was more potent than a CL isolate in promoting IFN-γ and TNF-α production. This finding lends support for distinct intraspecies differences and argues for the role of intraspecies differences and host factors contributing to the clinical outcome following *L. braziliensis *infection.

## Competing interests

The author(s) declare that they have no competing interests.

## Authors' contributions

PTGL, OB and EMC participated equally in the study design, and PTGL and OB performed all the immunological dosages.

PTGL, AS, JLH and EMC drafted the manuscript.

EMC participated in the coordination of *Leishmania *patient evaluations.

PRLM, RPA, AS, ARJ, and LHG performed clinical evaluation of LC and LD patients.

AG isolated and cultivated *Leishmania *and prepared both *L. braziliensis *antigens.

## Pre-publication history

The pre-publication history for this paper can be accessed here:


